# Field trial of medicinal plant, *Bidens pilosa*, against eimeriosis in broilers

**DOI:** 10.1038/srep24692

**Published:** 2016-04-21

**Authors:** Cicero Lee-Tian Chang, Cheng-Ying Yang, Thangarasu Muthamilselvan, Wen-Chin Yang

**Affiliations:** 1 Department of Veterinary Medicine, College of Veterinary Medicine, National Chung Hsing University, Taiwan; 2 Agricultural Biotechnology Research Center, Academia Sinica, Taiwan; 3 Department of Aquaculture, National Ocean University, Keelung Chung, Taiwan; 4 Institute of Pharmacology, Yang-Ming University, Taiwan; 5 Department of Life Sciences, National Chung-Hsing University, Taichung 402, Taiwan

## Abstract

Eimeriosis is a severe protozoan disease in poultry. Because of increasing concern about drug residue and drug resistance with the use of anticoccidial drugs, natural products are emerging as an alternative and complementary approach to control avian eimeriosis. Our previous publication showed that feed supplemented with *B. pilosa* (BP) was effective at combating chicken eimeriosis in experimental settings. However, its efficacy against chicken eimeriosis under field conditions is not known. Here, we investigated the efficacy of BP against eimeriosis on an organic chicken farm. We found that feed supplemented with BP, at the dose of 0.025% of feed or more, significantly reduced *Eimeria* infection. This treatment increased body weight gain and reduced feed conversion ratio, leading to superior growth performance. It lowered morbidity/mortality rate, decreased oocysts per gram of feces and gut pathology and augmented the anticoccidial index. Collectively, these data demonstrated the potential of BP to control chicken eimeriosis on chicken farms. BP can, therefore, be used as an effective means to control eimeriosis.

Eimeriosis is caused by the protozoan parasites of the genus *Eimeria* including *E. acervulina, E. brunetti, E. maxima, E. necatrix, E. praecox, E. mitis*, *E. mivati, E. tenella* and *E. hagani*^1^. *Eimeria* species can invade and multiply in different regions of guts and, eventually, oocysts are excreted in stool. As a consequence, this infection can result in gut pathogenesis, poor growth performance, and morbidity and mortality in chickens^2^,^3^,^4^,^5^.

In recent years, natural products are being seriously considered as an alternative to prevent and/or therapeutically constrain eimeriosis in poultry as an alternative to anticoccidial chemicals and vaccines^6^,^7^,^8^,^9^. So far, 68 plants, including BP, have been shown to have anticoccidial properties in small-scale studies^6^,^7^,^8^,^9^. In these settings, the chickens were often reared in controlled environments and artificially challenged with certain pathogen(s). Thus, large-scale field trials are required to better assess the efficacy of these products on chicken farms where different environmental factors (climate, temperature, humidity, *etc*.) and biological factors (farming stress, fecal-to-oral transmission, pathogen species/compositions/titer, *etc*.) exist. However, no such field trials have been conducted on plant-derived products, which may preclude bringing these products to the commercial market^6^.

*B. pilosa* (BP), an Asteraceae plant, grows worldwide. It has been promoted as a potherb and medicine for humans^10^. This plant has been reported to treat over 41 categories of disease such as protozoan infection, bacterial infection, gut disorders, immune disorders, *etc.*^11^. Over 200 phytochemicals have been identified from BP, including aliphatics, flavonoids, terpenoids, phenylpropanoids, aromatics, porphyrins and other compounds. The medicinal properties of BP may be attributed to its constituent compounds^11^. In terms of its anticoccidial activities, BP was originally reported for its use as a medicinal herb to treat malaria, a human coccidial disease^12^,^13^. Its *in vitro* efficacy suggested that BP and its active compounds are good therapeutic agents for malaria^14^. Polyacetylenes and flavonoids from BP were proposed as the active compounds responsible for its antimalarial activity^13^. However, the anti-coccidial mechanism of BP and the active constituents still remain elusive. These findings prompted us to test the effect of BP on *Eimeria*, another coccidial parasite in chickens. More recently, we have shown that BP reduced infection and drug resistance of *Eimeria* in chickens at the laboratory scale^9^. Further, BP increased beneficial bacteria species and decreased harmful bacteria species in the guts of chickens, probably accounting for part of its anticoccidial action^15^. In this study, to further evaluate its anticoccidial efficacy under intensive farming conditions, a field trial of BP was conducted on an organic chicken farm. Growth performance, such as body weight and feed conversion ratio (FCR) was compared in different groups of chickens. In parallel, excreted oocysts, type of stool, morbidity/mortality rate, gut lesions, and the anticoccidial index of the chickens were investigated.

## Results

### Effect of BP on culling rate of chickens in a field trial

Our previous publication demonstrated that BP reduced *Eimeria* infection in chickens in laboratory settings^9^. These promising data prompted us to verify the anticoccidial potential of BP in a field situation. Prior to this field trial, a survey of the composition and abundance of *Eimeria* species in the chicken farm was conducted. Morphological and molecular characterization revealed that seven *Eimeria* species, *E. acervulina*, *E. brunetti*, *E. maxima*, *E. necatrix*, *E. praecox*, *E. mitis* and *E. tenella* were present on the farm (Sup. Fig. S1). Among these, *E. acervulina*, *E. mitis*, *E. necatrix*, *E. praecox*, and *E. tenella* appeared to be more abundant than the other species (Sup. Fig. S1).

**Figure 1:**
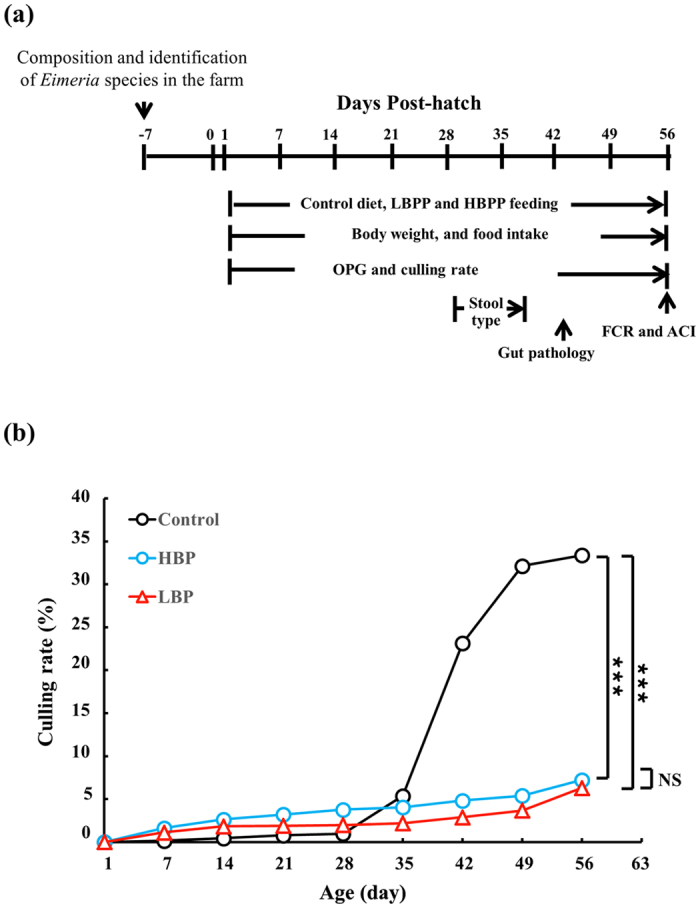
BP decreases culling rate of chickens in a field trial. (**a**) A scheme of the protocol for the field study. (**b**) Culling rate, the sum of sick and dead birds in each group. The birds fed with a standard diet (Control) or diet containing 0.05% BPP (HBPP) or 0.025% BPP (LBPP) from days 1 to 56 were monitored for morbidity and fatality, once a week, over 56 days. *P* values of more than 0.05 (NS) were considered not statistically significant and those less than 0.05 (*), 0.01 (**) and 0.001 (***) were considered statistically significant.

In this study, we performed a pilot field trial to assess the potential of BP to control eimeriosis on an organic chicken farm based on the protocol in Fig. 1a. The chickens were naturally infected with *Eimeria* species through fecal-to-oral transmission on the chicken farm. We first examined the effect of BP on the morbidity and mortality of chickens. The composition of standard diet and the diet supplemented with 0.025% BP product (BPP) (LBPP group) and 0.05% BPP (HBPP group) is indicated in Tables 1 and 2. We found that 33.4%, 6.3%, and 7.2% of the chickens in the Control, LBPP and HBPP groups, respectively, were culled because of morbidity and mortality (Fig. 1b). The data confirmed that in-feed treatment with BP could significantly reduce *Eimeria* species-implicated morbidity and mortality.

### Effect of BP on growth performance in chickens

Next, we evaluated the effect of BP on growth performance in chickens using body weight gain (BWG) and FCR parameters. We observed that, at the age of 56 days, Control chickens fed with standard diet reached an average BWG of 1773.0 ± 23.9 g. In contrast, those fed with LBPP and HBPP, had an average BWG of 2093.1 ± 34.2 g and 2033.1 ± 29.9 g, respectively (Table 3). Consistently, the chickens, fed with Control, LBPP and HBPP diets had an average FCR of 3.27 ± 0.04, 1.51 ± 0.02 and 1.93 ± 0.03, respectively (Table 3). Collectively, BWG and FCR data illustrated that in-feed treatment with BP improved growth performance in chickens at a large-scale farm.

### Effect of BP on gut pathology in chickens

Next, we examined gut pathology in the culled birds, aged 42 days, when the mortality/morbidity rate in Control birds surged. As shown in Table 4, when their guts were compared, Control chickens had higher gross lesion score (4.69 ± 0.40), than HBPP-fed chickens (1.42 ± 0.19) and LBPP-fed chickens (2.15 ± 0.18). Taking a closer look at different regions of the guts, Control chickens showed more profound damage to the duodena, jejuna/ilea and ceca than those fed with LBPP and HBPP as evidenced by their gross lesion scores (Table 4). Furthermore, we checked microscopic lesions in the guts of the same chickens as shown in Table 4 under a microscope. Accordingly, Control chickens had higher microscopic lesion score (15.67 ± 0.98), than HBPP-fed chickens (2.75 ± 0.54) and LBPP-fed chickens (5.92 ± 0.44) (Table 5). Further, Control chickens had more severe lesions in the duodena, jejuna/ilea and ceca than age-matched chickens fed with LBPP and HBPP (Table 5).

### Effect of BP on fecal oocyst excretion and percentage of bloody stools

**Figure 2:**
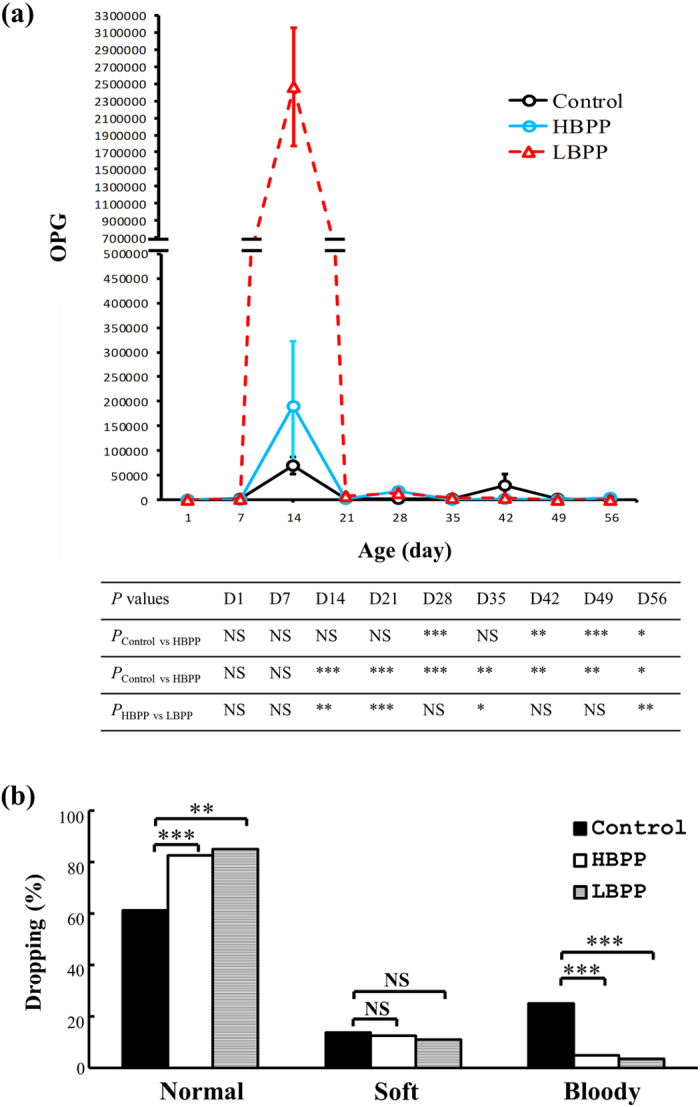
BP reduces OPG and percentage of bloody stools. OPG (**a**) and stool classification (**b**) in each group of chickens (Fig. 1a) were analysed once a week from day 1 to 56. The data on stool types of the chickens from day 28 to 35 are shown. *P* values of more than 0.05 (NS) were considered not statistically significant and those less than 0.05 (*), 0.01 (**) and 0.001 (***) were considered statistically significant.

In parallel, we analysed fecal oocyst excretion and types of stool. In this field test, the chickens encountered *Eimeria* oocysts present in their chicken houses via a natural transmission route from days 1 to 56. Their stools were analysed once a week over 56 days. First, we found that the fecal oocyst counts peaked every 2 weeks in 3 groups of chickens (Fig. 2a). This fluctuation could be attributed to re-infection, reflecting reports in previous publications^16^,^17^. Apparently, chickens in the LBPP group had much greater oocyst counts than those in the HBPP and Control groups as evidenced by the data of oocysts per gram of feces (OPG) (Fig. 2a). In-feed treatment with LBPP and HBPP seemed to control eimeriosis better than vehicle based on the fecal oocyst counts at later time points (Fig. 2a).

We also assessed the droppings in 3 groups of chickens. Stools from 3 groups of chickens were sampled and classified into normal, soft and bloody types (Fig. 2b). We observed that 25% of droppings from Control chickens were bloody compared to 4% and 5% of those in HBPP- and LBPP-fed chickens, respectively (Fig. 2b).

### Effect of BP on anticoccidial index

Anticoccidial index (ACI), a function of body weight gain (BWG), survival, gut lesion and fecal oocyst count has been used as an index to evaluate the anticoccidial activity of drugs. The Control diet had an ACI value of 83.1 (Control, Table 6). In contrast, HBPP and LBPP had ACI values of 197.7 and 188.5, respectively (HBPP and LBPP, Table 6). The ACI data collectively showed that LBPP and HBPP had stronger anticoccidial activities than the Control diet in the field situation.

Overall, the data confirmed that BP effectively constrained chicken eimeriosis in a large-scale field trial.

## Discussion

Eimeriosis has been a major cause of poor performance and loss of productivity in poultry farming. In view of the unmet need for anticoccidial agents^1^,^18^,^19^, botanicals are evolving as an alternative and complementary strategy to combat eimeriosis in poultry^6^,^7^,^8^,^9^. However, this strategy has been limited by lack of reliable field trials on the plant-related products. Thus, to date, very few products have entered the commercial market. Here, we report a pilot trial on the efficacy of BP in controlling eimeriosis following natural infection in an organic chicken farm housing 14,300 birds. Amazingly, our field trial data revealed that the prophylactic use of BP as a feed additive effectively reduced morbidity and death in organic chicken farming as shown by culling rate (Fig. 1). In line with the reduced morbidity/fatality, BP significantly decreased OPG (Fig. 2a), bloody stools (Fig. 2b), gut damage (Tables 4 and 5), and FCR (Table 3) and increased BWG (Table 3) and ACI (Table 6) in chickens.

In this field trial, anticoccidials and disinfectants were not used to kill oocysts, which are the source of natural infection with *Eimeria* species. However, this strategy led to different titers of oocysts in different chicken houses. For instance, the oocyst titer in the LBPP group appeared much higher than that in the HBPP and Control groups (Fig. 2). In spite of this drawback, 0.025% of BP in chicken feed was sufficient to control eimeriosis in field situations where there were 2.5 million OPG (day 14, LBPP, Fig. 2a) and multiple *Eimeria* species (Sup. Fig. S1) were present. These results suggest the superiority of BP, in terms of dose, to most botanicals published elsewhere^6^,^7^,^8^,^9^. However, down titration of the BP dose will be needed to seek its optimum dose prior to commercialization. On the test farm, the prevalence of eimeriosis in chickens was estimated to be 25% to 35% based on bloody stools (Fig. 2a) and culling rate (Fig. 1b). In-feed treatment with BP reduced this prevalence by over 20% (Fig. 1b), which is enough to counteract economic loss caused by *Eimeria* infection, not to mention the benefit of the additional body weight gain, FCR and shortened growth period of chickens (Table 3 and unpublished observation). Although numerous chickens were tested on a single farm in this study, a multiple-farm field trial will be required in order to assess the impact of the environment on the effectiveness of BP.

Overall, our data prove the concept that both high and low doses of BP are superior to a Control diet. Further, we observed that a high dose of BP (HBPP) had better clinical outcomes than a low dose of BP (LBPP), including with regard to gut pathology (Tables 4 and 5) and ACI values (Table 6). However, this observation of the relationship between dose and effect does not hold true in the case of culling rate, OPG and FCR. The discrepancy may be due to the different titers of oocysts in the 3 chicken houses. Another interesting observation was that, albeit fluctuating, OPG peaked by the age of 14 days in chickens and decreased later. This phenomenon was seen in chickens with natural infection^6^,^16^,^17^,^20^. BP seemed to fail to directly kill *Eimeria* species based on OPG and bloody stools. It is not hard to image that this incomplete eradication may be an advantage since the remaining live oocysts at low titer can serve as a vaccine to boost host immunity against *Eimeria* dissemination into the brood. Of note, the anticoccidial mode of action of BP needs to be further addressed and is under way. First, the data in a previous publication^9^ and this work suggest that BP interferes with but does not kill *Eimeria* species. Second, BP contributes to growth performance and anticoccidial control in chickens plausibly via modulation of gut microbiota including increased probiotics and decreased detrimental bacteria^15^. The compounds in BP that are active against eimeriosis have not yet been identified. However, polyacetylenes and flavonoids have been reported to possess antimalarial activity^13^. Whether or not both classes of compounds also contribute to the anticoccidial action of BP needs to be verified. Clearly, BP can exert its anticoccidial action via targeting *Eimeria* species and gut bacteria in chickens as described previously^6^,^7^,^8^. Taken together with our earlier publications^9^,^15^, our data illustrate the applicability of BP for prophylactic use to control eimeriosis in conventional and organic chicken farming.

## Methods

### Plant preparation and processing and feed formulation

BP product, BPP, used in the study, was purchased from a local company (Chun-Yueh Biomedical Technology, Taiwan) and processed according to the GMP guidelines as previously published^9^. Briefly, the BPP was made from a powder of the whole-plant BP. For its use as an in-feed agent, BPP was premixed with a standard chicken diet (Uni-President Enterprises, Taiwan) at a ratio of 1:10 in a premixer (Uni-President Enterprises, Taiwan), followed by mixing at a ratio of 1:100 (0.05% BPP) or 1:200 (0.025% BPP). Cytopiloyne, a polyacetylene isolated from BP, was used as an index compound for quality control of the supplementation procedure and batch consistency using high pressure liquid chromatography as published^9^. The ingredients of the standard diet (Table 1), approximate composition, gross energy of the standard diet, and the standard diet containing BP (Sup. Table S1) were analysed by the company according to the association of analytical communities guidelines.

### Animal husbandry and natural infection in the field trial

A field trial was carried out at a commercial organic farm in Miaoli, Taiwan. All animal experiments were performed according to the guidelines of National Chung-Hsin University, Taiwan. All the experimental protocols were approved by the Institutional Animal Care and Use Committee of National Chung-Hsin University (Protocol no. 103–34). A total of 14,300 naked-neck chickens were assigned to three groups, with one group per house. There were 5,000 birds in the Control group, 4,500 birds in the high-dose BP product (HBPP) group and 4,800 birds in the low-dose BP product (LBPP) group (Table 2). The chickens in the Control group were reared on a standard diet from day 1 to day 56 and those in the HBPP and LBPP groups were reared on a standard diet supplemented with 0.05% BPP and 0.025% BPP, respectively. The animals were naturally infected via a fecal-to-oral route in anticoccidial chemical-free surroundings. Prior to the field trial, the *Eimeria* species and compositions were examined by microscopy and polymerase chain reaction (PCR) using primer sets specific for *Eimeria* species (Sup. Fig. S1).

### Measurement of BWG, FCR, OPG, culling rate, gut pathology, ACI and stool classification

The experimental protocol is described in Fig. 1. The body weight, feed intake, OPG and culling rate in each group of chickens were monitored at the indicated intervals. The culling rate of the chickens was a summation of dead chickens and morbid chickens, as defined by sick bird appearance. BWG was obtained by the difference in body weight of the sampled chickens over a specified period of time. FCR was obtained by the ratio of feed intake to body weight gain of the sampled chickens. OPG was obtained by collection of sampled stools from chickens housed at the indicated time points as described elsewhere^9^,^21^. Briefly, 10 fresh stools in 8 areas of each chicken house were collected, weighed and suspended in water. Following filtration and centrifugation, the oocysts were suspended in saturated salt water and counted. Average oocyst number in the stools of each group was obtained. The same chicken droppings, 80 samples per group, were classified into normal, soft and bloody types and scored at the indicated time points. ACI was calculated based on the following formula, ACI = [relative body weight gain (RBWG, %) + survival rate (SR, %)] – [lesion score index (LSI) + oocyst count index (OI)], as previously published^22^,^23^.

For gut pathology, the 42-day-old chickens, 26 chickens per group, were sacrificed and their intestines and ceca were collected for further examination. As published^22^, gross lesion scores in duodena, jejuna/ilea and ceca were obtained. Briefly, gross lesions in the guts were scored based on a scale of 0 to 4:0, normal tissue without gross lesions (0% blood in feces); 1, few scattered petechiae on gut wall (25% blood in feces); 2, numerous petechiae (50% blood in feces); 3, extensive hemorrhage (75% blood in feces), and 4, extensive hemorrhage with dark color in guts (>75% blood in feces) or dead birds. Furthermore, microscopic lesions were scored as described previously^24^. Briefly, the guts were fixed with formalin, embedded in paraffin, and stained with hematoxylin and eosin. The location of cecal lesions and mucosal histology were examined. The distribution of *Eimeria* infection along the observed gut segment was graded on a scale of 0 to 4:0, no *Eimeria* in any field; 1, *Eimeria* in one field; 2, *Eimeria* in two fields; 3, *Eimeria* in three fields and 4, *Eimeria* in all four fields. The severity score in mucosae was graded as follows: 0, *Eimeria* in 0% of villi; 1, *Eimeria* in <25% of villi; 2, *Eimeria* in 25 to 50% of villi; 3, *Eimeria* in 51 to 75% of villi; 4, *Eimeria* in >75% of villi. The microscopic lesion score is the sum of grades (0 to 4) found in 12 sections per gut.

### Statistical analysis

Data from the field trial are represented as mean ± standard error of the mean (SEM). Pearson’s chi-square test was used to determine a significant difference in mortality rate between treatment and control groups. Two-way analysis of variance (ANOVA) was employed to determine a significant difference in BWG and FCR using the GLM procedure of SAS system. Data on lesion scores were analysed by chi-square test. *P* values of less than 0.05 were considered statistically significant.

## Additional Information

**How to cite this article**: Chang, C. L.-T. *et al.* Field trial of medicinal plant, *Bidens pilosa*, against eimeriosis in broilers. *Sci. Rep.* **6**, 24692; doi: 10.1038/srep24692 (2016).

## Supplementary Material

Supplementary Information

## Figures and Tables

**Table 1:** Ingredients and composition of chicken feed used in the field trial.

S. N.	Basal ingredient	SD %
1	Yellow corn	63.5
2	Soybean meal	16
3	Full fat soybean,	10
4	Fish meal	3.5
5	Wheat bran	3
6	Soybean oil	1.2
7	Calcium carbonate	1.0
8	Dicalcium phosphate	1.1
9	Salt	0.4
10	Lysine	0.2
11	Vitamin premix	0.02
12	Mineral premix	0.08
Total		100

S.N.: serial number; SD: standard diet.

**Table 2:** Experimental design for each group of chickens in the field trial.

S. N.	Group	Number of birds^a^	Diet/Treatment	Bird age (days)
1	Control	5000	SD	1–56
2	HBPP	4500	SD + 0.05% BPP	1–56
3	LBPP	4800	SD + 0.025% BPP	1–56

^a^Birds were divided into 3 groups. Each group was fed with a standard diet (SD) (Control group) or SD in combination with a high dose of BPP (HBPP) or a low dose of BPP (LBPP) from days 1 to 56. The number of chickens in each group is indicated.

**Table 3:** BWG and FCR of the birds in the field trial.

Parameters	*P* values		Control	HBPP	LBPP
	*Pa*^3^	*Pb*^4^			
BWG (g)^1^	<0.001	<0.001	1773.0 ± 23.9	2033.3 ± 29.9	2093.1 ± 34.2
FCR^2^	<0.001	<0.001	3.27 ± 0.04	1.93 ± 0.03	1.51 ± 0.02

^1^The chickens were the same as in Table 2. Their body weight gain (BWG) was obtained based on the following formula: BWG = body weight on day 56 minus body weight on day 1.

^2^FCR = (feed intake)/(BWG).

^3^*Pa* values from ANOVA test performed to assess the statistical significance between Control and HBPP groups.

^4^*Pb* values from ANOVA test performed to assess the statistical significance between Control and LBPP groups.

**Table 4:** Gross lesion scores in the guts of chickens in the field trial.

Group^2^	Gross lesion score (Mean rank)^1^			Lesion scores (Mean rank)^1,6^
	Duodenum^3^	Jejunum/Ileum^4^	Cecum^5^	
Control	1.69 ± 0.14 (49.77)	1.04 ± 0.14 (50.52)	1.96 ± 0.25 (61.31)	4.69 ± 0.40 (60.60)
HBPP	1.07 ± 0.12 (32.73)	0.15 ± 0.09 (24.12)	0.19 ± 0.12 (28.27)	1.42 ± 0.19 (21.83)
LBPP	1.19 ± 0.11 (36.00)	0.81 ± 0.15 (43.87)	0.15 ± 0.07 (28.92)	2.15 ± 0.18 (36.08)
Chi-square^7^	10.57	22.98	46.92	41.17
Probability^8^	<0.005	<0.001	<0.001	<0.001

^1^Gross lesion of the guts of the same chickens, aged 42 days, as in Table 2 was examined and scored as described in the Materials and Methods section. Data are expressed as mean ± SEM (Mean rank).

^2^The three groups of chickens received the treatment shown in Table 2.

^3,4,5^Different regions were examined in the intestines and ceca. These regions are the most common infection sites of *E. acervulina*, *E. maxima* and *E. tenella*, respectively.

^6^A sum of lesion scores in the intestine and cecum of each chicken, aged 42 days.

^7^Chi-square in df = 2 and Probability <0.05 which means it must be bigger than 5.99.

^8^*P* values from the Kruskal-Wallis test performed to assess the statistical significance between Control and HBPP or LBPP groups.

**Table 5:** Microscopic lesion scores in the guts of chickens fed a standard diet alone or in combination with BP.

Group^2^	Microscopic lesion score (Mean rank)^1^			Lesion scores (Mean rank)^1,6^
	Duodenum^3^	Jejunum/Ileum^4^	Cecum^5^	
Control	4.67 ± 0.51 (30.25)	3.33 ± 0.54 (26.25)	7.67 ± 0.14 (30.50)	15.67 ± 0.98 (30.50)
HBPP	0.50 ± 0.23 (10.75)	0.67 ± 0.28 (10.67)	0.67 ± 0.28 (6.83)	2.75 ± 0.54 (7.83)
LBPP	0.83 ± 0.17 (14.50)	1.83 ± 0.39 (18.58)	3.25 ± 0.22 (18.17)	5.92 ± 0.44 (17.17)
Chi-square^7^	24.47	15.17	31.31	28.38
Probability^8^	<0.001	<0.001	<0.001	<0.001

^1^Microscopic lesion of the guts of the same chickens, aged 42 days, as in Table 2 was examined and scored as described in the Materials and Methods section. Data are expressed as mean ± SEM (Mean rank).

^2^The three groups of chickens received the same treatment as in Table 2.

^3,4,^^5^Different regions were examined in the intestines and ceca. These regions are the most common infection sites of *E. acervulina*, *E. maxima* and *E. tenella*, respectively.

^6^A sum of lesion scores in the intestine and cecum of each chicken, aged 42 days.

^7^Chi-square in df = 2 and Probability <0.05 which means it must be bigger than 5.99

^8^*P* values from the Kruskal-Wallis test were used to assess the statistical significance between the Control and HBPP or LBPP groups.

**Table 6:** ACI values in each group of chickens in the field trial.

Group	RBWG (%)	SR (%)	LSI	OI	ACI
Control	100	70	46.9	40	83.1
HBPP	123.1	93.4	14.2	4.6	197.7
LBPP	119.7	97	21.5	6.7	188.5

ACI values are calculated based on the formula described in the Materials and Methods section. RBWG (%) = 100× [(BWG of treatment group)/(BWG of Control group)]; SR (%) = 100× [(the number of living chickens)/(total number of chickens per group)]; LSI = 10× (lesion score per group) and OI = 100 × 0.4× [(oocyst counts per group/oocyst counts in Control group)].

## References

[b1] Chapman H. D. *et al.* A selective review of advances in coccidiosis research. Adv. Parasitol. 83, 93–171 (2013). 23876872 10.1016/B978-0-12-407705-8.00002-1

[b2] Castanon J. I. History of the use of antibiotic as growth promoters in European poultry feeds. Poult. Sci. 86, 2466–2471 (2007). 17954599 10.3382/ps.2007-00249

[b3] Orengo J. *et al.* Evaluating the efficacy of cinnamaldehyde and Echinacea purpurea plant extract in broilers against Eimeria acervulina. Vet. Parasitol. 185, 158–163 (2012). 21996002 10.1016/j.vetpar.2011.09.024

[b4] Serratosa J. *et al.* Residues from veterinary medicinal products, growth promoters and performance enhancers in food-producing animals: a European Union perspective. Rev. Sci. Tech. 25, 637–653 (2006). 17094703

[b5] Williams R. Anticoccidial vaccines for broiler chickens: pathways to success. Avian Pathol. 31, 317–353 (2002). 12396335 10.1080/03079450220148988

[b6] Abbas R., Colwell D. & Gilleard J. Botanicals: An alternative approach for the control of avian coccidiosis. World’s Poul Sci J. 68, 203–215 (2012).

[b7] Bozkurt M., Giannenas I., Küçükyilmaz K., Christaki E. & Florou-Paneri P. An update on approaches to controlling coccidia in poultry using botanical extracts. Br. Poult. Sci. 54, 713–727 (2013). 24397508 10.1080/00071668.2013.849795

[b8] Quiroz-Castañeda R. E. & Dantán-González E. Control of avian coccidiosis: future and present natural alternatives. BioMed Res. Intl. 2015 (2015). 10.1155/2015/430610 PMC4346696 25785269

[b9] Yang W. *et al.* Effect of *Bidens pilosa* on infection and drug resistance of *Eimeria* in chickens. Res. Vet. Sci. 98, 74–81 (2015). 25440995 10.1016/j.rvsc.2014.11.002

[b10] Young P. H., Hsu Y. J. & Yang W. C. *Bidens pilosa* and its medicinal use. Drug plants. II 28, 411–426 (2010).

[b11] Bartolome A. P., Villasenor I. M. & Yang W. C. *Bidens pilosa* L. (Asteraceae): Botanical Properties, Traditional Uses, Phytochemistry, and Pharmacology. Evid. Based Complement. Alternat. Med. 2013, 340215 (2013). 23935661 10.1155/2013/340215 PMC3712223

[b12] Brandão M. G., Krettli A. U., Soares L. S., Nery C. G. & Marinuzzi H. C. Antimalarial activity of extracts and fractions from *Bidens pilosa* and other Bidens species (Asteraceae) correlated with the presence of acetylene and flavonoid compounds. J. Ethnopharmacol. 57, 131–138 (1997). 9254115 10.1016/s0378-8741(97)00060-3

[b13] Oliveira F. Q., Andrade-Neto V., Krettli A. U. & Brandão M. G. New evidences of antimalarial activity of *Bidens pilosa* roots extract correlated with polyacetylene and flavonoid. J. Ethnopharmacol. 93, 39–42 (2004). 15182902 10.1016/j.jep.2004.03.026

[b14] V. F. A.-N. *et al.* Antimalarial activity of *Bidens pilosa* L. (Asteraceae) ethanol extracts from wild plants collected in various localities or plants cultivated in humus soil. Phytother. Res. 18, 634–639 (2004). 15476304 10.1002/ptr.1510

[b15] Chang C. L. *et al.* Beneficial Effect of *Bidens pilosa* on Body Weight Gain, Food Conversion Ratio, Gut Bacteria and Coccidiosis in Chickens. Plos One 11, e0146141 (2016). 26765226 10.1371/journal.pone.0146141 PMC4713076

[b16] Al-Badri R. & Barta J. R. The kinetics of oocyst shedding and sporulation in two immunologically distinct strains of *Eimeria maxima*, GS and M6. Parasitol. Res. 111, 1947–1952 (2012). 22828932 10.1007/s00436-012-3041-4

[b17] Lunden A. *et al.* *Eimeria* infections in litter-based, high stocking density systems for loose-housed laying hens in Sweden. Br. Poult. Sci. 41, 440–447 (2000). 11128384 10.1080/713654973

[b18] De Gussem M. Coccidiosis in poultry: review on diagnosis, control, prevention and interaction with overall gut health. The 16th European Symposium on Poultry Nutrition, Strasbourg, France. Oxford, UK: The Centre for Agriculture and Bioscience International (2007, August 26).

[b19] Haug A., Gjevre A.-G., Thebo P., Mattsson J. G. & Kaldhusdal M. Coccidial infections in commercial broilers: epidemiological aspects and comparison of *Eimeria* species identification by morphometric and polymerase chain reaction techniques. Avian Pathol. 37, 161–170 (2008). 18393094 10.1080/03079450801915130

[b20] Almeida G. F. *et al.* The effects of combining *Artemisia annua* and *Curcuma longa* ethanolic extracts in broilers challenged with infective oocysts of *Eimeria acervulina* and *E. maxima*. Parasitology. 141, 347–355 (2014). 24553078 10.1017/S0031182013001443

[b21] Holdsworth P. *et al.* World Association for the Advancement of Veterinary Parasitology (WAAVP) guidelines for evaluating the efficacy of anticoccidial drugs in chickens and turkeys. Vet. Parasitol. 121, 189–212 (2004). 15135859 10.1016/j.vetpar.2004.03.006

[b22] Johnson J. & Reid W. M. Anticoccidial drugs: lesion scoring techniques in battery and floor-pen experiments with chickens. Exp. Parasitol. 28, 30–36 (1970). 5459870 10.1016/0014-4894(70)90063-9

[b23] Wang Z., Shen J., Suo X., Zhao S. & Cao X. Experimentally induced monensin-resistant *Eimeria tenella* and membrane fluidity of sporozoites. Vet. Parasitol. 138, 186–193 (2006). 16524664 10.1016/j.vetpar.2006.01.056

[b24] Goodwin M. A., Brown J. & Bounous D. I. Use of microscopic lesion scores, gross lesion scores and oocyst count scores to detect *Eimeria maxima* in chickens. Avian Pathol. 27, 405–408 (1998). 18484020 10.1080/03079459808419359

