# Supplementary material for: Field trial of medicinal plant, Bidens pilosa, against eimeriosis in broilers
Source: Sci Rep. 2016 Apr 21;6:24692. doi: 10.1038/srep24692 (PMC4838822; doi:10.1038/srep24692)
Supplement: Supplementary Information [file srep24692-s1.pdf]

Supplementary Information

Field trial of medicinal plant, Bidens pilosa, against eimeriosis in broilers

Cicero Lee-Tian Chang[1], Cheng-Ying Yang[2,3], Thangarasu Muthamilselvan[2]* and Wen-Chin Yang[2,3,4,5]*

S1A

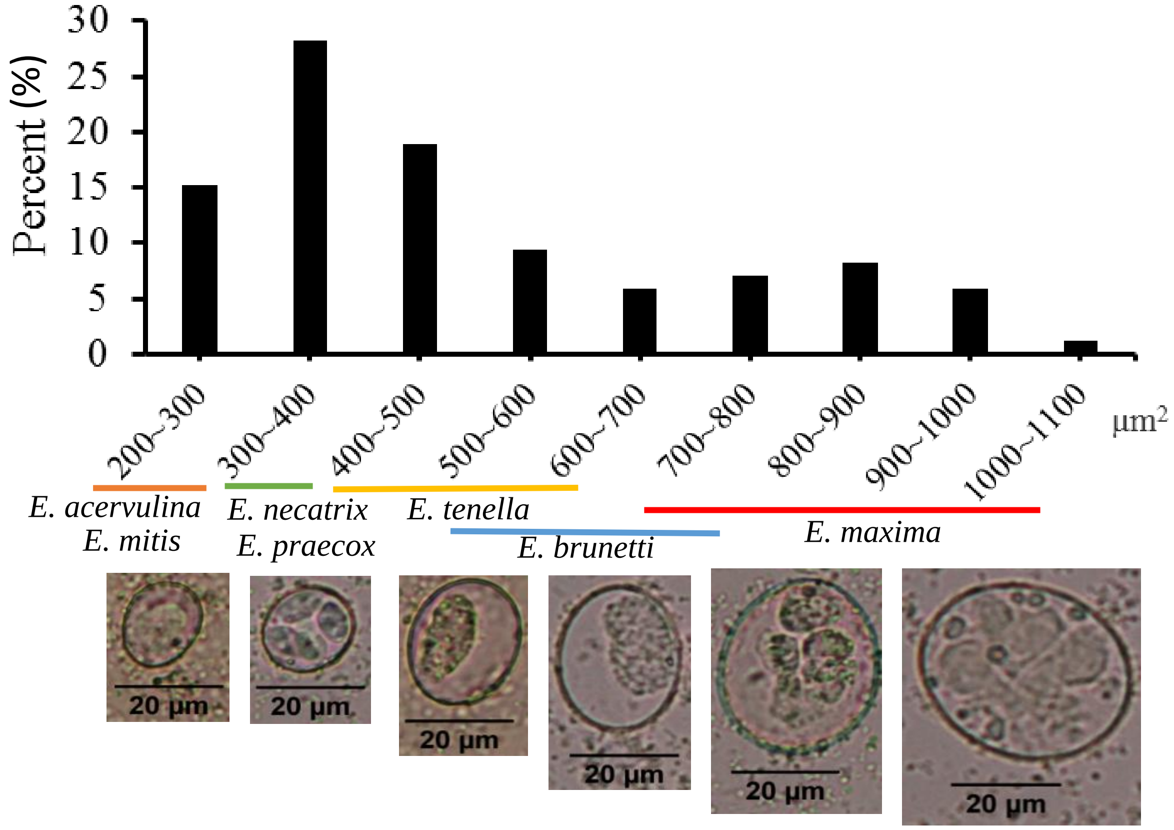

S1B

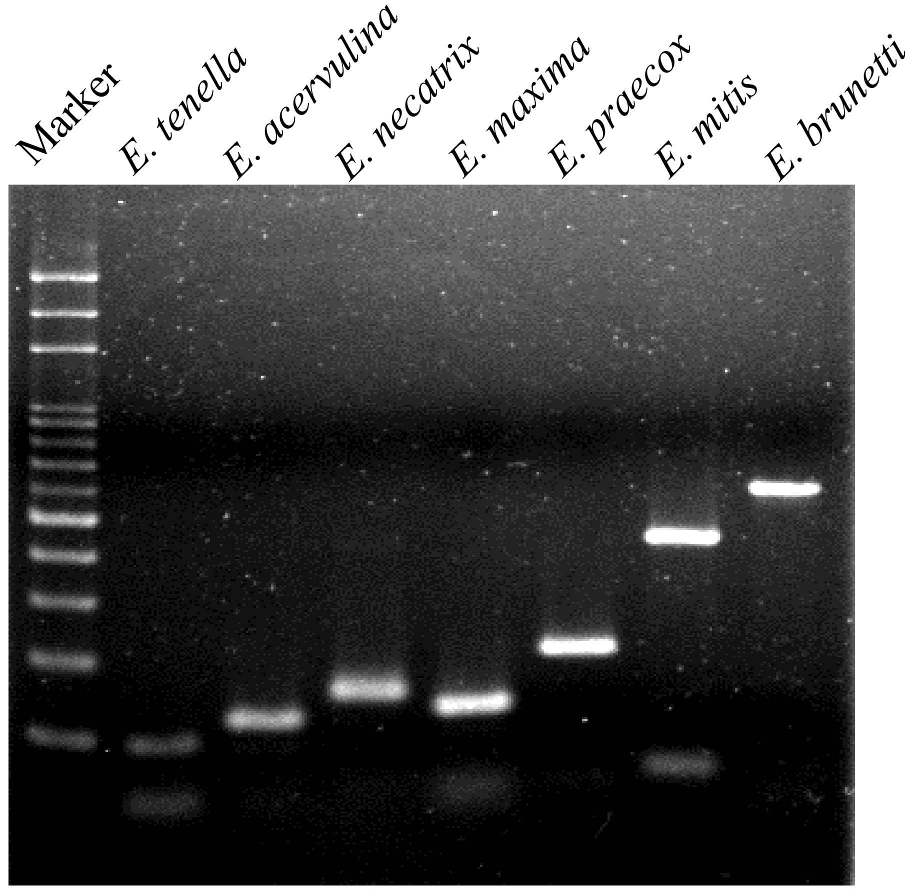

S1C

Species	Base pairs	Primer set
E. tenella	121 bp	TCGTCTTTGGCTGGCTATTC
		CAGAGAGTCGCCGTCACAGT
E. acervulina	124 bp	CTCGCGTGTCAGCACTACAT
		GATACGCTGCTTTGCCTTTC
E. necatrix	141 bp	AACGCCGGTATGCCTCGTCG
		GTACTGGTGCCAACGGAGA
E. maxima	138 bp	TCGTTGCATTCGACAGATTC
		TAGCGACTGCTCAAGGGTTT
E. praecox	278 bp	CATCGGAATGGCTTTTTGAAAGCG
		GCATGCGCTAACAACTCCCCTT
E. mitis	460 bp	AGTCAGCCACCAGTAGAGCCAATATTT
		AGTCAGCCACAAACAAATTCAAACTCTAC
E. brunetti	626 bp	TGGTCGCAGAACCTACAGGGCTGT
		TGGTCGCAGACGTATATTAGGGGTCTG

Sup. Fig. S1. Characterization of *Eimeria* species on the poultry farm. Feces were collected from the poultry farm before the field trial. (A) The oocysts were collected from stool samples, examined under a microscope and counted. Their composition and photographs are shown. (B-C) DNA from the oocysts (A) was extracted. The DNA was used as a template to perform polymerase chain reaction in the presence of 7 primer sets of *Eimeria* species (C). The PCR products and DNA markers were analysed using DNA gel electrophoresis. Their representative image is indicated (B).

Supplementary Table S1. Proximate composition and gross energy of the feed used in the study.

	Percentage of *B. pilosa*[a] in diets[a]		
	0% BP	0.025% BP	0.05% BP
Crude protein (%)	20.80	20.75	20.70
Crude fat (%)	7.39	7.37	7.35
Ash (%)	4.54	4.53	4.52
Crude fiber (%)	2.61	2.60	2.60
Carbohydrate (%)	52.79	52.91	53.03
Gross Energy (kcal/kg)	3100	3092	3085
CP[b] (μg/kg)	0	14.3	28.5

[a]Composition of standard diet (0% BP) and standard diet containing 0.025% and *B. pilosa extract* (0.05% BP) *B. pilosa extract*, respectively.

[b]Cytopioyne (CP) content of different diets was determined based on HPLC chromatography.
